# Developmental changes in cerebral NAD and neuroenergetics of an antioxidant compromised mouse model of schizophrenia

**DOI:** 10.1038/s41398-023-02568-2

**Published:** 2023-08-05

**Authors:** Radek Skupienski, Pascal Steullet, Kim Q. Do, Lijing Xin

**Affiliations:** 1 Center for Psychiatric Neuroscience, Department of Psychiatry, Lausanne University Hospital (CHUV), Prilly, Switzerland; 2 CIBM Center for Biomedical Imaging, Lausanne, Switzerland; 3 Animal Imaging and Technology, Ecole Polytechnique Fédérale de Lausanne (EPFL), Lausanne, Switzerland

**Keywords:** Schizophrenia, Biomarkers

## Abstract

Defects in essential metabolic regulation for energy supply, increased oxidative stress promoting excitatory/inhibitory imbalance and phospholipid membrane dysfunction have been implicated in the pathophysiology of schizophrenia (SZ). The knowledge about the developmental trajectory of these key pathophysiological components and their interplay is important to develop new preventive and treatment strategies. However, this assertion is so far limited. To investigate the developmental regulations of these key components in the brain, we assessed, for the first time, in vivo redox state from the oxidized (NAD^+^) and reduced (NADH) form of Nicotinamide Adenine Dinucleotide (NAD), energy and membrane metabolites, inhibitory and excitatory neurotransmitters by ^31^P and ^1^H MRS during the neurodevelopment of an SZ animal model with genetically compromised glutathione synthesis (*gclm*-KO mice). When compared to age-matched wild type (WT), an increase in NAD^+^/NADH redox ratio was found in *gclm*-KO mice until early adulthood, followed by a decrease in full adults as observed in patients. Especially, in early postnatal life (P20, corresponding to childhood), levels of several metabolites were altered in *gclm*-KO mice, including NAD^+^, NAD^+^/NADH, ATP, and glutamine + glutamate, suggesting an interactive compensation for redox dysregulation between NAD, energy metabolism, and neurotransmission. The identified temporal neurometabolic regulations under deficits in redox regulation provide insights into preventive treatment targets for at-risk individuals, and other neurodevelopmental disorders involving oxidative stress and energetic dysfunction.

## Introduction

Schizophrenia (SZ) is a neurodevelopmental syndrome, affecting ∼0.4% of the population, which is arising from both genetic and environmental factors [1]. Although the disease is characterized by an increased dopamine release in the striatum (upregulation of the mesolimbic pathway) and a reduced release in the prefrontal cortex (downregulation of the mesocortical pathway) [2], other pathophysiological processes have been implicated: dysfunction during development in NMDAR-mediated signaling, neuroimmune regulation, or mitochondrial function appear to initiate “vicious circles” centered on redox dysregulation/oxidative stress, leading to anomalies of parvalbumin interneurons (PVI) and excitatory/inhibitory imbalance, at the basis of cognitive deficit [3–5]. Additionally, defects in essential metabolic processes for energy supply [6] and membrane function [7] have also been implicated in SZ.

Glutathione (GSH), which is one of the main cellular antioxidants, has been reported to be reduced in the brain of some patients with schizophrenia [8–10]. Proper redox homeostasis relies on the tight reciprocal interactions between the antioxidant systems and the energy metabolism which produces reactive oxygen species [11]. The energy metabolism is dependent on many redox enzymatic reactions involving co-agonist redox couples, such as NAD^+^ (oxidized) and NADH (reduced) forms of nicotinamide adenine dinucleotide (NAD). They act as cofactors in bioenergetic pathways and play a fundamental role in redox reactions, such as glycolysis, oxidative phosphorylation, free radical detoxification, and superoxide production [12]. The cellular oxidoreductive state can be estimated by the NAD redox ratio (NAD^+^/NADH). NAD^+^ is also involved as a co-substrate in many other biologically relevant processes, including calcium homeostasis, immunological functions, carcinogenesis, cell death, and gene expression [13]. Moreover, the ratio NAD^+^/NADH has recently been shown to be decreased in the frontal lobe of patients with SZ [14], suggesting a redox imbalance in SZ. However, it is unclear how key pathophysiological components, including NAD redox status, cerebral energy metabolism, excitatory and inhibitory neurotransmission, membrane metabolism, and their interplay, unfold from childhood to the onset of the disease. Such knowledge is important for developing new preventive and treatment strategies.

In this study, we aim to assess how a redox dysregulation caused by a deficit in GSH as seen in subsets of patients impacts NAD^+^/NADH, energy metabolism, excitatory and inhibitory metabolites along the postnatal development period. Previously, we demonstrated the feasibility of measuring NAD^+^/NADH, energy, and membrane metabolites during mouse brain development using phosphorus magnetic resonance spectroscopy (^31^P-MRS) [15] at 14.1 T, which is challenging because of the low sensitivity due to the small brain size. Here, we pushed towards the characterization of developmental alterations in a mouse model related to SZ, which displays reduced GSH synthesis (*gclm*-KO) leading to several characteristic features of the disease, including impaired inhibitory GABAergic parvalbumin-positive interneurons (PVI) in numerous brain regions [16], neuroinflammation, compromised mitophagy [17], white matter and oligodendrocyte anomalies [18, 19] together with altered behaviors [20]. *Gclm*-KO mouse phenotype is characterized by a 60–70% decrease in GSH and a slightly lower weight as compared to wild-type control (WT) [21]. In addition, its blood glucose and liver glycogen are lower, and its basal systemic metabolic rate is elevated [22]. This mouse also depicts a redox imbalance leading to increased oxidative stress together with a greater vulnerability to external stresses [23].

Therefore, we focused on the measurement of frontal NAD^+^ and NADH content, energy metabolites (ATP, phosphocreatine and inorganic phosphate), metabolites associated with neurotransmission (*γ*-aminobutyrate acid), glutamate and glutamine (both being associated with neurotransmission and metabolism as energy source) by ^31^P and ^1^H MRS during the development of *gclm*-KO and WT mice to investigate the developmental trajectory of these key components. Then, the association between them was assessed to investigate whether the mechanistic links between these processes along brain development may be relevant for the pathophysiology of SZ.

## Materials and Methods

### Study plan

**Fig. 1:**
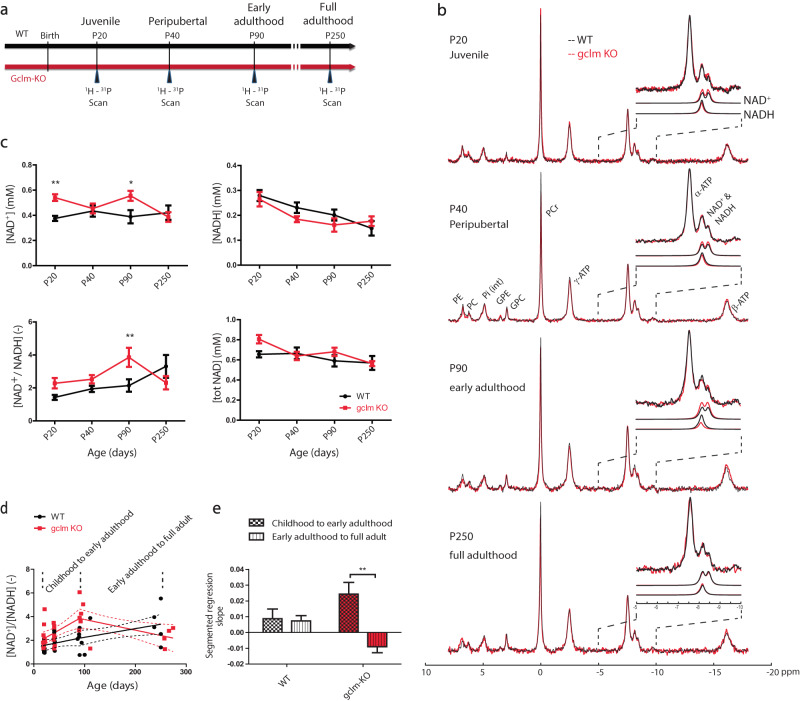
Developmental changes of cerebral NAD content and NAD^+^/NADH ratio in *gclm*-KO and WT mice. Changes of NAD content and redox ratio in *gclm*-KO and WT mice during brain development. **a** Study scheme presenting the different time-points and their corresponding developmental stages at which mice were scanned. **b** Summed ^31^P-MRS spectra (see the zoom on α-ATP and NAD spectral region) where the developmental changes of NAD^+^ and NADH can be visually observed along with age (from P20 to P250) in WT and *gclm*-KO mice. **c** NAD^+^, NADH, NAD^+^/NADH, and total NAD levels from P20 to P250 in WT and *gclm*-KO mouse brain. Significant increase of NAD^+^ at P20 and P90, and NAD^+^/NADH at P90 as compared to age-matched WT. At P250, a decrease of NAD^+^/NADH is observed in the *gclm*-KO as compared to P90, while in WT a stable NAD^+^ and decrease in NADH lead to an increase in RR. Switch of NAD^+^/NADH regulation in *gclm*-KO mice. **d** shows a stable increase of NAD^+^/NADH in WT, and an increase of NAD^+^/NADH until early adulthood followed by a decrease during adulthood in *gclm*-KO. **e** Slopes of the segmented regression depict the difference between WT and *gclm*-KO mice (**p = 0.005), where a switch in NAD^+^/NADH occurs after early adulthood (P90) in *gclm*-KO, but not in WT.

To have an overview of the critical time point in neurodevelopment implicated in schizophrenia, a cohort of mice was scanned at different ages corresponding to different developmental stages (Fig. 1a): Postnatal day 20 (P20, the end of the suckling period); P40 (the puberty period); P90 (the end of adolescence or early adulthood usually corresponding to the first appearance of psychotic episode in SZ) and finally P250 (mature adult) [24–26].

### Animal model

*Gclm*-KO mouse model was generated and kindly provided by TP. Dalton (Cincinnati University, Ohio). *Gclm*-KO and WT mice born in the local animal facility were housed in ventilated cages on a 12-h light-dark cycle at room temperature of 20-22 °C with 50-60% humidity. After weaning at P21, a minimum of 2 and a maximum of 5 animals were kept per cage. Tap water and regular chow were provided *ad libitum*. The experiments were conducted with a cohort of males/females, aged from 20 to 250 days with a bodyweight of 7–40 g. Respective animal numbers, age, genotype, males/females were as follows: P20, WT (5 m/6 f) *gclm*-KO (6 m/6 f); P40, WT (4 m/5 f) *gclm*-KO (5 m/3 f); P90, WT (4 m/5 f) *gclm*-KO (4 m/5 f); P250, WT (4 m/1 f) *gclm*-KO (3 m/1 f). Detailed description of animal preparation and the sample size for each metabolite at each data point (Table 1S) are available in the supplemental material. All animal procedures were performed according to federal guidelines and were approved by the Swiss cantonal veterinarian authorities.

### In vivo MR Spectroscopy

All MR experiments were performed on a 14.1 T small animal scanner with a 26 cm horizontal bore (Magnex Scientific, Abingdon, United Kingdom), equipped with a 12 cm internal diameter gradient coil insert (400 mT/m, 120 µs) and a DirectDrive console interface (Agilent Technologies, Palo Alto, CA, USA). Radiofrequency transmission/reception was achieved using a homebuilt three-channel surface coil, namely two single-turn loops (10 mm diameter) 90° geometrically decoupled, quadrature ^1^H coil with one loop linearly polarized ^31^P coil (10 mm diameter). Fast spin-echo multi-slice images were first acquired for voxel positioning using the following parameters: repetition time of 3.3 s, echo time of 43.24 ms, echo train length of 8, echo spacing of 10.81 ms, the field of view of 20 ×20 mm, matrix size 128 × 128, slice thickness 0.4 mm, 35 slices and 2 averages. Local shimming in the volume of interest (VOI) was achieved using 1st- and 2nd-order shims with FAST(EST)MAP [27].

Water suppressed ^1^H MR spectra were acquired in the anterior cingulate cortex (ACC) from a volume of 5.76 µL (0.9 × 4 × 1.6 mm, supplemental material, Fig. S1) using a SPECIAL sequence with an echo time of 2.75 ms, repetition time of 4 s, and 240 scans (30 × 8 blocks). The transmitter frequency was set on the water resonance for the acquisition of water spectra (8 scans) for metabolites quantification and eddy current correction. For normalization of the ^1^H MRS, the brain water content was measured at P20, P40, and P90 using the weight difference between freshly removed and fast dissected brain structures, and their residue after lyophilization (Supplemental material, Fig. S2). The water content at a later stage (P250) was assumed to be stable and the same as that at P90.

^31^P MR spectra were acquired in the fronto-dorsal part of the brain using a 3D-ISIS localization in combination with a pulse-acquire sequence composed by an adiabatic half passage pulse (500 µs pulse width) with the transmitter offset was set on NAD^+^ (-8.3 ppm). The following measurement parameters were used: voxel size 90 µL (2.5 × 6 × 6 mm^3^) at P20 and P40, 122.5 µL (2.5 × 7 × 7 mm^3^) at P90 and P250, TR = 5 s, 1600 averages (100 blocks of 16 averages), 12 kHz spectral width and 4096 complex points. To ensure a consistent tissue contribution across the different developmental stages, the VOI was increased at P90 and P250 to account for the slight brain enlargement when compared with P20 and P40.

### Spectral quantification

Frequency drift and phase variation were corrected prior to the summation of all block spectra. The ^1^H and ^31^P metabolite concentrations were determined with LCModel (Stephen Provencher Inc., Oakville, Ontario, Canada). Cramér-Rao lower bound (CRLB) was used as an exclusion criterion for all ^1^H and ^31^P data and the cutoff was set at a maximum of 30%.

^1^H basis set containing a measured macromolecule spectrum and simulated metabolite spectra was used together with the unsuppressed water signal measured from the same VOI as an internal reference for the absolute quantification of metabolites. The following 21 metabolites were included in the analysis: acetate (Ace), alanine (Ala), ascorbate (Asc), aspartate (Asp), creatine (Cr), *γ*-aminobutyrate (GABA), glucose (Glc), glutamine (Gln), glutamate (Glu), glycine (Gly), glycerophosphocholine (GPC), glutathione (GSH), myo-inositol (Ins), lactate (Lac), N-acetyl-aspartate (NAA), N-acetyl-aspartylglutamate (NAAG), phosphocholine (PCho), phosphocreatine (PCr), phosphoethanolamine (PE), scyllo-inositol (Scyllo), taurine (Tau).

^31^P metabolite concentrations were normalized using PCr levels obtained from the ^1^H experiments. Apodization with a 10 Hz exponential function was applied to all spectra prior to spectral quantification. The ^31^P metabolites quantification was performed with a basis-set prepared with simulated ^31^P spectra including PCr, *α*-ATP, *β*-ATP, *γ*-ATP, Pi^int^ (intracellular inorganic phosphate), Pi^ext^ (extracellular inorganic phosphate), PE (phophothanolamine), PC (phosphocholine), GPC (glycerophosphocholine), GPE (glycerophosphoethanolamine), MP (membrane phospholipid), NADH, NAD^+^, with respective linewidths [15]. With resonances overlapping with NADH and NAD^+^, uridine diphosphate glucose (UDPG) is a phosphorylated sugar intermediate in biochemical reactions. Due to the low concentration and sensitivity of UDPG in the mouse brain, additional analysis was performed with summed age pooled spectra with the inclusion of UDPG in the basis-set to quantify its level and to evaluate its effect on NAD quantification results. Physiological parameters (pH^int^ and free [Mg^2+^]) were calculated from chemical shift differences between ^31^P metabolites (details of the calculation can be found herein [15]).

### Statistical analysis

All statistical analyses were performed in Graphpad Prism 5 (GraphPad Software, inc.), Matlab (R2017a), or R (R Core Team, 2019). All variables were tested by two-way ANOVA using age and genotype as a fixed factor for the comparison between groups (*gclm*-KO vs. WT). The results of two-way ANOVA are summarized in Table 2S. In the case of significant interaction, *post hoc* tests were further performed. The effect of age or genotype was *post hoc* investigated between groups using the Bonferroni correction for multiple comparisons (multiplicity adjusted *P*-values are computed). To evaluate the presence of regular increase or decrease with age that would not be detected by ANOVA, linear regressions along age were performed. Correlations between all metabolites were measured using two-tailed Pearson correlation with 95% of the confidence interval and reported using the correlation coefficients R^2^. Significance of the correlation coefficient difference between genotype groups was tested using the Fisher r-to-z transformation. The results are presented as the mean and standard error of the mean unless otherwise stated. When exact *p*-values are not provided, significant differences (*) were considered for *P* < 0.05, (**) for *P* < 0.01, (***) for *P* < 0.001 and (****) for *P* < 0.0001.

## Results

### NAD content during neurodevelopment

NAD content in mouse fronto-dorsal brain was monitored in vivo by ^31^P MRS at four time points corresponding to different postnatal developmental periods (Fig. 1, Table 1). The summed ^31^P MR spectra, from P20 to P250, illustrate the differences between WT and *gclm*-KO (Fig. 1b) together with the excellent sensitivity and spectral quality at 14.1 T. A zoom on the NAD spectral region allows us to visually recognize the temporal changes of NAD^+^ and NADH in both genotypes.

**Table 1:** Concentration and CRLBs of NAD^+^, NADH, total NAD, and RR at P20, P40, P90, and P250, respectively.

AGE [Days]	GENOTYPE	NAD^+^ [mM]	CRLB [%]	NADH [mM]	CRLB [%]	RR [-]	TOTAL NAD [mM]
**P20**	WT	0.38 ± 0.06	10 ± 1	0.28 ± 0.07	11 ± 2	1.43 ± 0.47	0.66 ± 0.10
	*Gclm*-KO	0.54 ± 0.08	9 ± 2	0.27 ± 0.09	14 ± 3	2.28 ± 0.99	0.81 ± 0.13
**P40**	WT	0.43 ± 0.13	9 ± 2	0.23 ± 0.06	12 ± 3	1.94 ± 0.56	0.67 ± 0.18
	*Gclm*-KO	0.46 ± 0.11	8 ± 1	0.18 ± 0.03	13 ± 2	2.53 ± 0.70	0.64 ± 0.12
**P90**	WT	0.39 ± 0.15	10 ± 3	0.20 ± 0.06	14 ± 3	2.15 ± 1.06	0.59 ± 0.16
	*Gclm*-KO	0.56 ± 0.12	8 ± 2	0.16 ± 0.07	19 ± 4	3.85 ± 1.53	0.68 ± 0.12
**P250**	WT	0.42 ± 0.13	9 ± 1	0.15 ± 0.06	19 ± 5	3.31 ± 1.55	0.57 ± 0.15
	*Gclm*-KO	0.39 ± 0.08	11 ± 1	0.18 ± 0.04	17 ± 3	2.31 ± 0.80	0.56 ± 0.05

^31^P MR spectra of individual animals demonstrated good spectral resolution (linewidth of PCr = 13–15 Hz) and good sensitivity (SNR = 30–50), which ensured the reliable quantification of the NAD signals with a mean CRLB of 9% for NAD^+^ and 15% for NADH. A significant difference between genotypes (two-way ANOVA with genotype and age as co-factors, *P* = 0.009) was observed for NAD^+^. However, age did not have a significant influence on NAD^+^, while an interaction between age and genotype (*P* = 0.044) was found. The increase of NAD^+^ was found in *gclm*-KO as compared to WT at P20 (*P* < 0.01) and at P90 (*P* < 0.05) (Fig. 1c). For NADH, no significant difference was observed between genotypes, but there was a strong age effect (*P* = 0.0002, Fig. 1c), with NADH levels decreasing with age. Similarly, total NAD content did not depict any significant differences between genotypes but an age effect was observed (*P* = 0.020).

NAD^+^/NADH redox ratio was shown to be affected by genotype (two-way ANOVA, *P* = 0.044) and age (*P* = 0.006). An interaction between age and genotype was also found (*P* = 0.019). Elevated NAD^+^/NADH was found in *gclm*-KO compared to WT at P90 (*P* < 0.01). Further analysis using one-way ANOVA test on each genotype depicted a significant difference between age groups (WT, *P* = 0.006; *gclm*-KO, *P* = 0.030), and a post-test for linear trend showed a significant increase with age only in WT (*P* = 0.0006, R^2^ = 0.341, slope = 0.292). *Post hoc* test showed a NAD^+^/NADH increase between P20 and P250 (*P* < 0.01) in WT and between P20 and P90 (*P* < 0.05) in *gclm*-KO. Beyond P90, NAD^+^/NADH started to decrease in *gclm*-KO. Thus, to further characterize this observation, a segmented regression was performed (Fig. 1d). The segmented regression analysis (R Core Team, 2019), (corrected for repeated measures, age, and sex) depicted a switch at P90 in *gclm*-KO, with a positive slope from childhood to early adulthood and a negative slope from early adulthood to full adult (difference between both slopes, *P* = 0.005, Fig. 1e). In WT, no differences were found between these two slopes.

### Energy metabolites (ATP, PCr, and Pi)

**Fig. 2:**
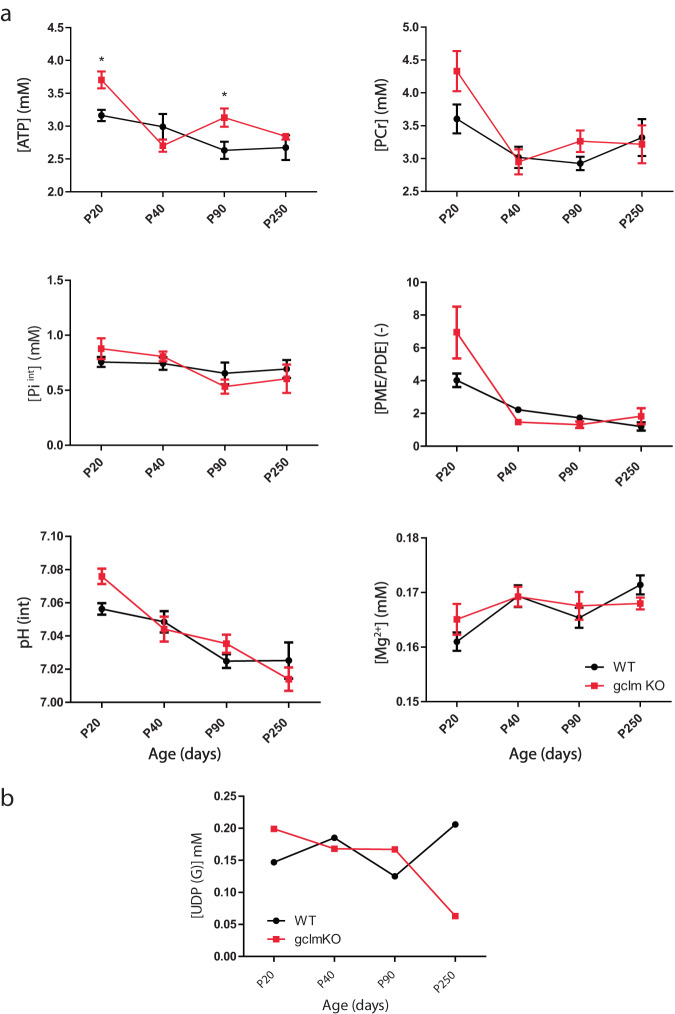
Brain developmental changes in ATP, phosphocreatine (PCr), Intracellular phosphate (Pi^int^), pH, [Mg^2+^], phosphoester ratio PME/PDE, and UDP(G) in WT and *gclm*-KO mice. Changes of [ATP], [PCr], [Pi ^int^], pH, [Mg^2+^], PME/PDE and UDP(G) from postnatal day P20 to P250 in the brain of WT and *gclm*-KO mice. **a** *Gclm*-KO mice demonstrated significant increases in ATP at P20 and P90 relative to WT mice. All values are shown as mean ± SEM and significant differences are derived from the *post hoc* Bonferroni correction test for multiple comparisons. *P < 0.05. **b** Levels of UDP(G) were obtained from the pooled summed spectra, respectively at P20, P40, P90, and P250. A stable level along development was observed in WT mice, while a decrease after P90 occurred in *gclm*-KO mice.

Genotype (*P* = 0.030), age (*P* < 0.0001) and the interaction between them (*P* = 0.010) had an influence on ATP levels. Significantly higher ATP level was observed in *gclm*-KO at P20 (*P* < 0.05) and at P90 (*P* < 0.05) in comparison to WT (Fig. 2a). In WT, the ANOVA did not depict significant differences between age groups for ATP, but a slight age-related decrease was proven by the linear trend (*P* = 0.018, *R*^2^ = 0.172, slope = −0.092) along with age. In *gclm*-KO, ATP was significantly different between age groups (*P* < 0.0001). Post-test revealed a decrease from P20 to P40 (*P* < 0.0001), P20 to P90 (*P* < 0.01) and P20 to P250 (*P* < 0.01). A decreased linear trend with age was also observed (*P* = 0.003, *R*^2^ = 0.163, slope = -0.108). A strong age effect was observed for PCr (two-way ANOVA, *P* < 0.0001); however, genotype difference was not detected. Pi^int^ did not differ between genotypes, but had a minor age effect (*p* < 0.05).

### Metabolites associated with excitatory and inhibitory (E/I) neurotransmission (Gln, Glu, and GABA)

**Fig. 3:**
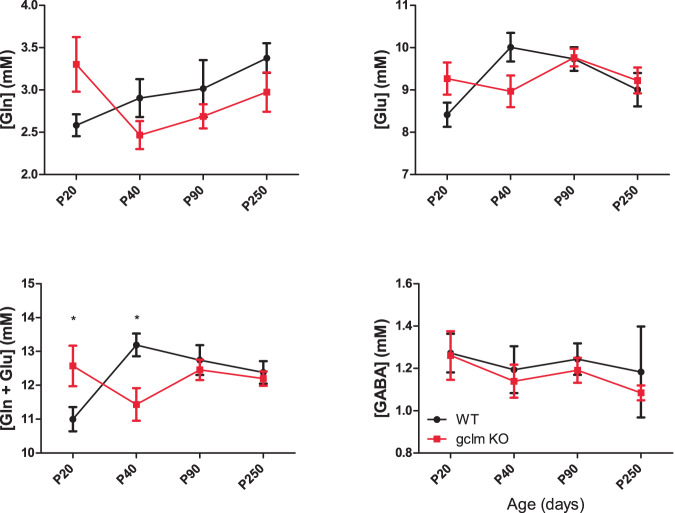
Concentrations (mM) of metabolite associated with excitatory and inhibitory (E/I) neurotransmission in *gclm*-KO and WT mouse brain during development. Concentrations of metabolites associated with neurotransmission including Glu, Gln, Glu + Gln, and GABA in *gclm*-KO and WT mouse brain during brain development. Strong trends of increases in Gln and Glu in *gclm*-KO at P20 lead to a significant increase of Glx (Gln + Glu). At P40, a significant reduction of Glx was observed in *gclm*-KO in comparison to WT.

No genotype difference was detected for Gln and Glu, but an age effect was found for Glu (*P* = 0.030). However, for both Gln and Glu, which are also both associated with energy metabolism, an interaction between age and genotype was observed (*P* = 0.028 and *P* = 0.029, respectively). Both metabolites demonstrated a trend towards higher levels at P20 and lower levels at P40 in *gclm*-KO as compared to WT (Fig. 3). Furthermore, the analysis of their sum (Gln + Glu) presented neither genotype nor age differences, but a strong interaction (*P* = 0.0015). *Post hoc* analysis showed significantly more elevated Gln + Glu at P20 (*P* < 0.05), and lower Gln + Glu at P40 (*P* < 0.05) in *gclm*-KO in comparison to age-matched WT (Fig. 3). In WT, significant changes were observed with age (*P* < 0.0008), with post-hoc tests revealing a decrease of Glu + Gln between P20 and P40 (*P* < 0.001), and between P20 and P90 (*P* < 0.05). In contrast, age did not influence Gln + Glu in *gclm*-KO mice. Finally, the concentrations of GABA did not differ between genotype and along with age.

### Association between NAD content, neurotransmission, and energy metabolism

**Fig. 4:**
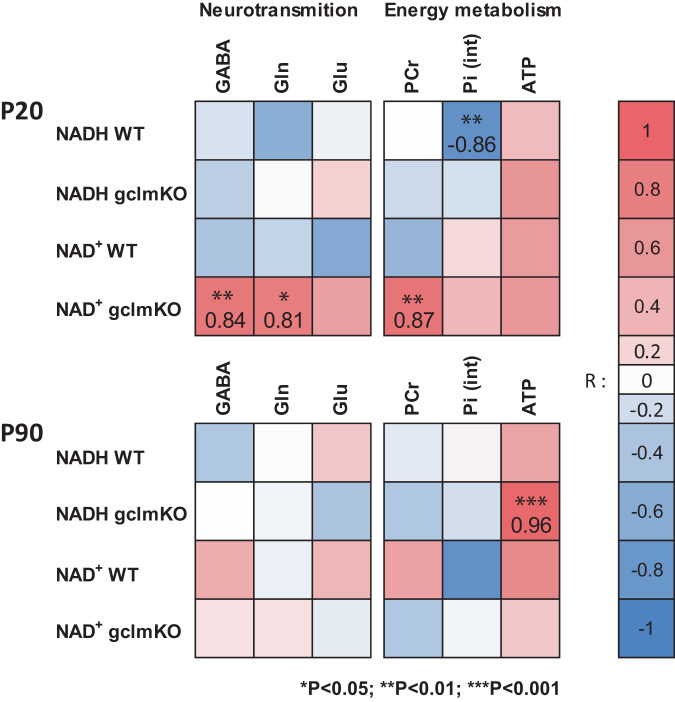
Associations between NAD content, E/I neurotransmission, and energy metabolism. Correlation matrix depicts in *gclm*-KO and WT mouse the links between NAD content (NAD^+^ and NADH), neurotransmission, and energy metabolism at P20 and P90. At the early developmental period (P20), NAD^+^ is strongly associated with GABA, Gln, and PCr in *gclm*-KO animals, while NADH is exclusively associated with inorganic phosphate in WT mice. In early adulthood (P90), *Gclm*-KO mice showed a strong positive association between their ATP and NADH levels. Heatmaps show the correlation coefficients. *P < 0.05, **P < 0.01, ***P < 0.001.

To investigate the interplay between NAD redox regulation, energy metabolism, and E/I neurotransmission, the correlations between the different components were analyzed at P20 and P90 which corresponded, in our study, to pivotal points of brain development (Fig. 4). P20 corresponds to the end of the suckling period after which a drastic change in alimentation happens together with the weaning. P90 matches with early adulthood in humans when the first psychotic episode occurs, and thus seems to represent a crucial transition phase from prodromal alterations to acute psychosis. At P20, NAD^+^ positively correlated with GABA (*P* = 0.009, *R*^2^ = 0.704), Gln (*P* = 0.016, *R*^2^ = 0.650) and PCr (*P* = 0.005, *R*^2^ = 0.755) in *gclm*-KO mice, but not in WT mice. NADH negatively correlated with Pi (*P* = 0.001, *R*^2^ = 0.739) only in WT. At P90, NADH positively correlated with ATP (*P* = 0.0006, *R*^2^ = 0.923) only in *gclm*-KO mice. Using the Fisher r-to-z transformation, we found significant differences in the correlations between NAD^+^ and GABA, Glu, Gln, and PCr at P20 when comparing *gclm*-KO and WT animals.

### Membrane metabolites

Phosphomonoesters (PME) (i.e. phosphoetanolamine and phosphocholine) that reflect membrane synthesis, and phosphodiesters (PDE) (i.e. glycero-phosphoetanolamine and glycero-phosphocholine) that reflect membrane degradation, were measured by ^31^P MRS (Fig. S3). Thus, the ratio (PME/PDE) represents the balance between membrane synthesis and degradation. No difference between genotype was detected in the PME/PDE ratio by two-way ANOVA, but a strong age effect was observed (*P* < 0.0001, Fig. 2a).

### Uridine diphosphate glucose (UDPG)

UDPG is a phosphorylated nucleotide sugar intermediate of cell polysaccharide synthesis. Due to the low level of UDPG, we summed all individual animal spectra at each age to improve the SNR. A three-fold decrease of UDPG was observed at P250 in *gclm*-KO relative to WT animals (Fig. 2b, Table 3S). For the exploratory analysis of UDPG at the single animal level, we observed a significant interaction between age and genotype (*p* = 0.038), but no significant main effects of age or genotype (Table 4S). *Post hoc* analysis using Bonferroni correction revealed a significant decrease in UDPG levels in *gclm*-KO mice compared to WT mice at P250 (*p* < 0.05), which is consistent with the results obtained from the summed spectra (Fig. 2b).

### pH and [Mg^2+^]

pH and magnesium concentration [Mg^2+^] were also assessible by ^31^P MRS to monitor brain physiology. For pH, no difference between genotypes was detected by two-way ANOVA, but a strong age effect was observed (*P* < 0.0001, Fig. 2a). For [Mg^2+^], no difference was found between WT and gclm-KO, but an age effect was observed (*P* < 0.018).

## Discussion

With the sensitivity and spectral resolution enhancement at the ultra-high magnetic field (14.1 T), using both in vivo ^31^P and ^1^H-MRS, we have identified, for the first time, the effects of a redox dysregulation due to genetically lower GSH levels on postnatal development of the NAD redox state, energy homeostasis, membrane metabolism, levels of excitatory and inhibitory neurotransmitters and their interactions in the prefrontal brain region. In *gclm*-KO mice, most alterations were observed during early life (at P20). This includes upregulation of NAD^+^, increases in Gln + Glu, and higher levels of the key energy metabolite ATP. In addition, NAD^+^ was positively correlated with GABA, Gln and PCr in *gclm*-KO at P20 but not in WT mice. At the end of adolescence/early adulthood (P90), the brain of *gclm*-KO mice also displayed concomitantly elevated levels of NAD^+^, redox ratio, and ATP as compared to age-matched WT mice. Noteworthy, developmental changes in NAD redox ratio was observed for *gclm*-KO mice. Thus, NAD redox ratio increased with age (from P20 to P90) with the highest value at P90, and then sharply dropped in older full adulthood *gclm*-KO, suggesting a shift from oxidative stress toward a more reductive state at adulthood. Such decline of NAD^+^/NADH in adult *gclm*-KO mice was similar to the one displayed by SZ patients [14], implying that a compensatory mechanism associated with the increase of NAD^+^ started to fail during adulthood as a consequence of a long-lasting, exacerbated oxidative stress.

### Developmental adaptation of NAD^+^ under oxidative stress

The ∼70% decrease of brain endogenous antioxidant GSH in *gclm*-KO mice during brain development (Fig. S4) induces oxidative stress in the ACC [23] and other sub-cortical structures [16]. In the ACC of *gclm*-KO, the levels of oxidative stress, as quantified by 8-oxo-2’-deoxyguanosine, increased with age from P20 to P180 [16]. High levels of 8-oxo-2’-deoxyguanosine appeared to be present in mitochondria and were accompanied by compromised mitophagy [17]. In *gclm*-KO brains, the upregulation of NAD^+^ (at P20 and P90) and NAD redox ratio (with a peak value at P90) may reflect an adaptation to genetically induced oxidative stress. The sharp decrease of NAD redox ratio in *gclm*-KO mice older than P90 could indicate that such compensatory mechanism via upregulation of NAD^+^ declines in adult *gclm*-KO brains, leading to an exacerbation of the deleterious effects of oxidative stress such as a decreased number of PVIs in the ACC after the age of P90 [16]. Noteworthy, although PVI maturation appears to be delayed at early postnatal period (P5–P10) [16], it reaches the level of WT mice from peripuberty to young adulthood (P20–P90) in the ACC, which may partially be attributed to redox homeostasis compensation associated to the NAD^+^ upregulation up to P90 [28].

In response to redox dysregulation, NAD^+^-consuming enzymes like poly(ADP-ribose) polymerases (PARPs) and sirtuins can be activated as a protective mechanism against oxidative stress [29]. This heightened NAD^+^ consumption can stimulate the upregulation of the NAD^+^ salvage pathway, which helps replenish NAD^+^ levels. Furthermore, redox dysregulation can induce the expression and activity of enzymes involved in NAD^+^ biosynthesis. For instance, under oxidative stress conditions, one notable enzyme involved in NAD^+^ biosynthesis, called nicotinamide phosphoribosyltransferase (NAMPT), can be upregulated [30]. NAMPT facilitates the conversion of nicotinamide to nicotinamide mononucleotide (NMN), which serves as an intermediate in the NAD^+^ biosynthesis pathway. By upregulating NAMPT, cells can enhance NAD^+^ synthesis, thereby counterbalancing the increased NAD^+^ consumption resulting from redox dysregulation. Indeed, NAD^+^ has been shown to play a pivotal role in DNA repair and protein deacetylation. High levels of NAD^+^ may promote health and extend lifespan [31]. The exogenous promotion through supplementation in NAD^+^ precursor-like niacin [32], nicotinamide [33], nicotinamide mononucleotide [34], or nicotinamide riboside [35] has been shown to be effective in cell protection by suppressing the oxidative stress in a pathology associated with mitochondrial dysfunction [36].

### Association of NAD^+^ with GABA and Gln in *gclm*-KO mice at early developmental stage

Furthermore, although the GABA levels are not altered through development in *gclm*-KO, they are positively correlated with NAD^+^ at P20, a critical time window during which PVIs and brain circuitry undergo intense development and maturation. This suggests that the upregulation of NAD^+^ may be critically associated with the maintenance of the GABA levels in *gclm*-KO, to prevent deficits in the development of the inhibitory system. Meanwhile, the increase in NAD^+^ was also associated with an increase in Gln at P20. The last reaction step in the de novo synthesis and some of the NAD^+^ salvage pathways is the amidation of nicotinic acid adenine dinucleotide, which is achieved by the glutamine dependent NAD^+^ synthetase [37].

Taken together, NAD^+^ may serve as a potential preventive treatment target, especially for high-risk populations. Here, the assessment of NAD^+^ and NAD redox ratio has been limited to the prefrontal cortex. However, *gclm*-KO mice display oxidative stress and PVI deficits in a temporal and regional specific manner, with PVI anomalies appearing earlier in subcortical regions (i.e. thalamic reticular nucleus, globus pallidus, and hippocampus) than in the ACC [16]. Therefore, a further investigation of the regional and temporal alterations of NAD^+^ and NAD redox ratio and their association with PVI deficits would be extremely valuable to guide the preventive time window of NAD^+^ associated treatment.

### Energy metabolism alterations during neurodevelopment of *gclm*-KO mice

During early life, the brain undergoes extensive development and maturation, and high energy is required to form new membranes and myelin sheets around axons. As compared to age-matched WT, the brain of young *gclm*-KO mice (at P20) may undergo an exacerbated increase in metabolic activity as highlighted by high energy phosphate, ATP (Fig. 2a). Indeed, adult *gclm*-KO mice have a higher basal systemic oxygen consumption as compared to WT mice [22]. Kendig et al. observed an enhanced activity of mitochondrial complex 1 electron transport and an increase in mitochondrial respiration in the liver of *gclm*-KO mice. However, the situation might differ in the brain. Thus, we cannot exclude that the high ATP levels in the brain of gclm-KO mice at P20 indicate reduced energy demand as compared to age-matched WT mice due to the delayed maturation of the fast-spiking PVIs. However, in young adults, high ATP levels are also observed in brain of *gclm*-KO together with a lower weight, reduced plasma glucose and hepatic glycogen levels as compared to WT [38, 39] . This is consistent, among other possibilities, with an elevated oxidative phosphorylation. In any case, our data reveal an altered balance between ATP formation and use in cortex of *gclm*-KO mice. Of note, ATP was also suggested to have a protective effect against H_2_O_2_ induced oxidative damage [40].

In patients with SZ, neither ATP nor phosphocreatine (PCr) was consistently affected [41]. But a magnetization transfer experiment revealed that the reaction rate between PCr and ATP, catalyzed by creatine kinase, was decreased in the ACC of patients [42]. Here, we established a link between redox state and energy metabolism, however, the enzymatic activity measurement of creatine kinase and ATP synthase would provide additional light on ATP metabolism and would constitute a further step in the comprehension of the *gclm*-KO physiology. PCr correlates positively with NAD^+^ only in *gclm*-KO at P20, which suggests a potential adaptation of creatine kinase activity with the upregulation of NAD^+^.

Interestingly at P40, the energy metabolites do not differ in WT and *gclm*-KO mice, while reduced Gln + Glu levels are found in *gclm*-KO as compared to WT mice. This contrasts with the elevated Gln + Glu levels in P20 *gclm*-KO, as also observed previously [42]. Exaggerated synaptic pruning as well as the use of Gln and Glu as a substitute to power the TCA cycle [43–45] might explain potentially the drop in Gln + Glu in P40 *gclm*-KO mice. Glutamatergic metabolites appear to be elevated in the prodromal and early stages of schizophrenia [46], but unchanged or reduced below normal in chronic or medicated patients.

Overall, the level of high energy metabolite ATP depends on the balance between energy generation and utilization. The ATP levels alone cannot discern whether the elevated ATP observed in *gclm*-KO mice is a consequence of increased energy generation or reduced energy utilization. To gain further insights, future studies incorporating ^31^P magnetization transfer experiments, which can probe metabolic rates of ATP synthesis and utilization, will be essential for elucidating the underlying mechanisms and providing a more comprehensive understanding of ATP dynamics underlying redox dysregulation.

### UDP(G), glycogenesis, and extracellular matrix

UDP-glucose is the precursor for glycogenesis. In the brain of the *gclm*-KO mouse, the UDPG signal was drastically reduced at P250. Interestingly, adult *gclm*-KO mice displayed low levels of glycogen in their liver [38]. Likewise, cultured astrocytes from *gclm*-KO mice brains have lower glycogen levels, while its turnover is higher [39]. This further supports that the regulation of brain energy metabolism is altered together with reduced NAD redox ratio in fully adult *gclm*-KO. Note that oxidative stress challenge induced by tBHQ in human fibroblasts also causes a significant decrease in UDPG [47].

Recently, the UDPG signal was proposed to result not only from UDP-glucose, but also from other metabolites such as (by order of prevalence) UDP(G): UDP-N-Acetyl-glucosamine (UDP-GlcNAc); UDP-glucose(UDPGlc); UDP-N-Acetyl-galactosamine (UDP-GalNAc), and UDP-galactose(UDP-Gal) [48]. These molecules are used for the synthesis of the polysaccharide chains of the extracellular matrix that are made of alternating sugar/uronic acid and amino-sugars [49]. Therefore, the drop of UDPG signal in the brain of adult *gclm*-KO mice might reflect compromised integrity of the perineuronal net, the extracellular matrix enwrapping PVI, as a consequence of chronic oxidative stress. Noteworthy, the extracellular matrix and its homeostasis also appear to be abnormal in patients [47, 50]. The apparent decrease of UDP(G) in full adulthood of *gclm*-KO mice is intriguing, however, further investigation is required, as the spectral SNR achieved in small animal brains is still insufficient to establish which UDP(G) component is altered.

As a limitation in NAD quantification, the UDPG signal overlaps with NAD and has been shown to have an impact on NAD measurements [51]. Thus, the inclusion of UDPG in the quantification lowers preferentially the NADH content and leads to a higher NAD redox ratio. In the present study, similar results were observed for *gclm*-KO and WT mice on pooled age summed spectra. The NADH values were reduced and NAD redox ratio increased (Table. 3S). The decrease of the NAD redox ratio from P90 to P250 in *glcm*-KO remains nevertheless consistent with the results without the inclusion of UDPG and is even more pronounced with the inclusion of UDPG in the analysis (Table. 3S).

### Limitations

The objective of this study was to investigate the postnatal development period until adulthood. In the older age investigated in our study (P250), we have observed an early impact of aging on metabolites which is highly intriguing. As a future perspective, a comprehensive MRS time-lapse study of the mouse brain, fully encompassing the aging process, should be conducted to obtain a more comprehensive understanding across the entire lifespan.

The quantification of ^31^P metabolites is commonly performed using ATP, total phosphorus signal, or PCr obtained from ^31^P MRS as internal references. However, due to the variation of these reference signals during brain development, we opted to use the average PCr level determined by ^1^H MRS as an internal reference specific to each age and genotype group. The sensitivity of ^31^P is relatively low, necessitating a larger voxel size to obtain reasonable acquisition duration. On the other hand, in order to achieve excellent shimming and high spectral resolution for PCr measurement in ^1^H MRS, a smaller voxel is utilized. Consequently, a partial volume effect arises since ^1^H- and ^31^P-MRS were acquired from different sizes of volume of interest. This partial volume effect represents a limiting factor in our study. However, the quantification method does not impact the ratios between metabolites (e.g., NAD^+^/NADH). Furthermore, the quantification of pH and Mg^2+^ content relies on chemical shift differences, which remain unaffected by the spectral normalization method.

## Conclusion

This study reveals that deficits in redox regulation is associated with a dynamic neurodevelopmental trajectory, leading to specific temporal alterations in NAD redox state and neuroenergetics during the neurodevelopment. The upregulation in NAD^+^, and its highly associated regulations in energy metabolism and neurotransmission at the early developmental period, may be compensatory neuroprotective mechanisms under oxidative stress. This mechanistic insight holds great promise concerning potential preventive treatment targets for at-risk individuals.

### Supplementary information

Supplementary file

## References

[1] Owen MJ, Sawa A, Mortensen PB (2016). Schizophrenia. Lancet.

[2] Guzman F. The four dopamine pathways relevant to antipsychotics pharmacology**—**Psychopharmacology Institute. 2019. https://psychopharmacologyinstitute.com/publication/the-four-dopamine-pathways-relevant-to-antipsychotics-pharmacology-2096#%20References. Accessed 27 Aug 2020.

[3] Cannon TD (2015). How Schizophrenia develops: cognitive and brain mechanisms underlying onset of Psychosis. Trends Cogn Sci.

[4] Hardingham GE, Do KQ (2016). Linking early-life NMDAR hypofunction and oxidative stress in schizophrenia pathogenesis. Nat Rev Neurosci.

[5] Cuenod M, Steullet P, Cabungcal J-H, Dwir D, Khadimallah I, Klauser P (2021). Caught in vicious circles: a perspective on dynamic feed-forward loops driving oxidative stress in schizophrenia. Mol Psychiatry.

[6] Yang J, Chen T, Sun L, Zhao Z, Qi X, Zhou K (2013). Potential metabolite markers of schizophrenia. Mol Psychiatry.

[7] Mahadik SP, Evans DR (2003). Is schizophrenia a metabolic brain disorder? Membrane phospholipid dysregulation and its therapeutic implications. Psychiatr Clin.

[8] Do KQ, Trabesinger AH, Kirsten-Krüger M, Lauer CJ, Dydak U, Hell D (2000). Schizophrenia: glutathione deficit in cerebrospinal fluid and prefrontal cortex in vivo. Eur J Neurosci.

[9] Wang AM, Pradhan S, Coughlin JM, Trivedi A, DuBois SL, Crawford JL (2019). Assessing brain metabolism with 7-T proton magnetic resonance spectroscopy in patients with first-episode Psychosis. JAMA Psychiatry.

[10] Das TK, Javadzadeh A, Dey A, Sabesan P, Théberge J, Radua J (2019). Antioxidant defense in schizophrenia and bipolar disorder: a meta-analysis of MRS studies of anterior cingulate glutathione. Prog Neuropsychopharmacol Biol Psychiatry.

[11] Perkins DO, Jeffries CD, Do KQ (2020). Potential roles of redox dysregulation in the development of Schizophrenia. Biol Psychiatry.

[12] Ying W (2008). NAD+/NADH and NADP+/NADPH in cellular functions and cell death: regulation and biological consequences. Antioxid Redox Signal.

[13] Pollak N, Dölle C, Ziegler M (2007). Biochem J.

[14] Kim S-Y, Cohen BM, Chen X, Lukas SE, Shinn AK, Yuksel AC (2017). Redox Dysregulation in Schizophrenia Revealed by in vivo NAD+/NADH Measurement. Schizophr Bull.

[15] Skupienski R, Do KQ, Xin L (2020). In vivo 31 P magnetic resonance spectroscopy study of mouse cerebral NAD content and redox state during neurodevelopment. Sci Rep..

[16] Cabungcal J-H, Steullet P, Kraftsik R, Cuenod M, Do KQ (2019). A developmental redox dysregulation leads to spatio-temporal deficit of parvalbumin neuron circuitry in a schizophrenia mouse model. Schizophrenia Res.

[17] Khadimallah I, Jenni R, Cabungcal J-H, Cleusix M, Fournier M, Beard E (2021). Mitochondrial, exosomal miR137-COX6A2 and gamma synchrony as biomarkers of parvalbumin interneurons, psychopathology, and neurocognition in schizophrenia. Mol Psychiatry.

[18] Corcoba A, Steullet P, Duarte JMN, Van de Looij Y, Monin A, Cuenod M (2015). Glutathione deficit affects the integrity and function of the Fimbria/Fornix and anterior commissure in mice: relevance for Schizophrenia. Int J Neuropsychopharmacol.

[19] Monin A, Baumann PS, Griffa A, Xin L, Mekle R, Fournier M (2015). Glutathione deficit impairs myelin maturation: relevance for white matter integrity in schizophrenia patients. Mol Psychiatry.

[20] Kulak A, Duarte JMN, Do KQ, Gruetter R (2010). Neurochemical profile of the developing mouse cortex determined by in vivo 1H NMR spectroscopy at 14.1 T and the effect of recurrent anaesthesia. J Neurochem.

[21] das Neves Duarte JM, Kulak A, Gholam-Razaee MM, Cuenod M, Gruetter R, Do KQ (2012). N-Acetylcysteine normalizes neurochemical changes in the glutathione-deficient Schizophrenia mouse model during development. Biol Psychiatry.

[22] Kendig EL, Chen Y, Krishan M, Johansson E, Schneider SN, Genter MB (2011). Lipid metabolism and body composition in Gclm(−/−) mice. Toxicol Appl Pharmacol.

[23] Cabungcal J-H, Steullet P, Kraftsik R, Cuenod M, Do KQ (2013). Early-life insults impair parvalbumin interneurons via oxidative stress: reversal by N-acetylcysteine. Biol Psychiatry.

[24] Dutta S, Sengupta P (2016). Men and mice: relating their ages. Life Sci.

[25] Hagan C, D.V.M., Ph.D. When are mice considered old? The Jackson Laboratory. 2017. https://www.jax.org/news-and-insights/jax-blog/2017/november/when-are-mice-considered-old. Accessed 29 Jul 2020.

[26] Sengupta P (2013). The laboratory rat: relating its age with human’s. Int J Prev Med.

[27] Gruetter R, Tkác I (2000). Field mapping without reference scan using asymmetric echo-planar techniques. Magn Reson Med.

[28] Xie N, Zhang L, Gao W, Huang C, Huber PE, Zhou X (2020). NAD + metabolism: pathophysiologic mechanisms and therapeutic potential. Signal Transduct Target Ther.

[29] Johnson S, Imai S (2018). NAD+ biosynthesis, aging, and disease. F1000Res.

[30] Nishida T, Naguro I, Ichijo H (2022). NAMPT-dependent NAD+ salvage is crucial for the decision between apoptotic and necrotic cell death under oxidative stress. Cell Death Discov.

[31] Rajman L, Chwalek K, Sinclair DA (2018). Therapeutic potential of NAD-boosting molecules: the in vivo evidence. Cell Metab.

[32] Pirinen E, Auranen M, Khan NA, Brilhante V, Urho N, Pessia A (2020). Niacin cures systemic NAD+ deficiency and improves muscle performance in adult-onset mitochondrial myopathy. Cell Metab.

[33] Yang J, Klaidman LK, Nalbandian A, Oliver J, Chang ML, Chan PH (2002). The effects of nicotinamide on energy metabolism following transient focal cerebral ischemia in Wistar rats. Neurosci Lett.

[34] de Picciotto NE, Gano LB, Johnson LC, Martens CR, Sindler AL, Mills KF (2016). Nicotinamide mononucleotide supplementation reverses vascular dysfunction and oxidative stress with aging in mice. Aging Cell.

[35] Hong G, Zheng D, Zhang L, Ni R, Wang G, Fan G-C (2018). Administration of nicotinamide riboside prevents oxidative stress and organ injury in sepsis. Free Radic Biol Med.

[36] Zhu Y, Zhao K-K, Tong Y, Zhou Y-L, Wang Y-X, Zhao P-Q (2016). Exogenous NAD(+) decreases oxidative stress and protects H2O2-treated RPE cells against necrotic death through the up-regulation of autophagy. Sci Rep..

[37] Santos ARS, Gerhardt ECM, Moure VR, Pedrosa FO, Souza EM, Diamanti R (2018). Kinetics and structural features of dimeric glutamine-dependent bacterial NAD+ synthetases suggest evolutionary adaptation to available metabolites. J Biol Chem.

[38] Lavoie S, Steullet P, Kulak A, Preitner F, Do KQ, Magistretti PJ (2016). Glutamate cysteine ligase-modulatory subunit knockout mouse shows normal insulin sensitivity but reduced liver glycogen storage. Front Physiol.

[39] Lavoie S, Allaman I, Petit J-M, Do KQ, Magistretti PJ (2011). Altered glycogen metabolism in cultured astrocytes from mice with chronic glutathione deficit; relevance for neuroenergetics in schizophrenia. PLoS One.

[40] Lee YJ, Lee JH, Han HJ (2006). Extracellular adenosine triphosphate protects oxidative stress-induced increase of p21(WAF1/Cip1) and p27(Kip1) expression in primary cultured renal proximal tubule cells: role of PI3K and Akt signaling. J Cell Physiol.

[41] Yuksel C, Tegin C, O’Connor L, Du F, Ahat E, Cohen BM (2015). Phosphorus magnetic resonance spectroscopy studies in schizophrenia. J Psychiatr Res.

[42] Du F, Cooper AJ, Thida T, Sehovic S, Lukas SE, Cohen BM (2014). In vivo evidence for cerebral bioenergetic abnormalities in schizophrenia measured using 31P magnetization transfer spectroscopy. JAMA Psychiatry.

[43] Hosios AM, Hecht VC, Danai LV, Johnson MO, Rathmell JC, Steinhauser ML (2016). Amino acids rather than glucose account for the majority of cell mass in proliferating mammalian cells. Dev Cell.

[44] Reitzer LJ, Wice BM, Kennell D (1979). Evidence that glutamine, not sugar, is the major energy source for cultured HeLa cells. J Biol Chem.

[45] Stumvoll M, Perriello G, Meyer C, Gerich J (1999). Role of glutamine in human carbohydrate metabolism in kidney and other tissues. Kidney Int.

[46] Bustillo J, Rowland L, Mullins P, Jung R, Chen H, Qualls C (2010). 1H-MRS at 4 Tesla in minimally treated early schizophrenia. Mol Psychiatry.

[47] Fournier M, Ferrari C, Baumann PS, Polari A, Monin A, Bellier-Teichmann T (2014). Impaired metabolic reactivity to oxidative stress in early psychosis patients. Schizophr Bull.

[48] Ren J, Malloy CR, Sherry AD (2020). Quantitative measurement of redox state in human brain by 31P MRS at 7T with spectral simplification and inclusion of multiple nucleotide sugar components in data analysis. Magn Reson Med.

[49] Bosiacki M, Gąssowska-Dobrowolska M, Kojder K, Fabiańska M, Jeżewski D, Gutowska I et al. Perineuronal nets and their role in synaptic homeostasis. Int J Mol Sci. 2019. 10.3390/ijms20174108. 10.3390/ijms20174108 PMC6747153 31443560

[50] Pantazopoulos H, Katsel P, Haroutunian V, Chelini G, Klengel T, Berretta S Molecular signature of extracellular matrix pathology in schizophrenia. Eur J Neurosci. 2020. 10.1111/ejn.15009. 10.1111/ejn.15009 PMC8359380 33070392

[51] de Graaf RA, De Feyter HM, Brown PB, Nixon TW, Rothman DL, Behar KL (2017). Detection of cerebral NAD+ in humans at 7 T. Magn Reson Med.

