# Supplementary material for: Developmental changes in cerebral NAD and neuroenergetics of an antioxidant compromised mouse model of schizophrenia
Source: Transl Psychiatry. 2023 Aug 5;13:275. doi: 10.1038/s41398-023-02568-2 (PMC10404265; doi:10.1038/s41398-023-02568-2)
Supplement: Supplementary file 1 — Supplementary file [file 41398_2023_2568_MOESM1_ESM.pdf]

Supplementary Material

Developmental changes in cerebral NAD and neuroenergetics of an antioxidant compromised mouse model of schizophrenia

Radek Skupienski, Pascal Steullet, Kim Q. Do and Lijing Xin

Animal preparation

Animals were anesthetized with isoflurane (0.9 -1.2%) in a mixture of air and O_2 (50/50%). The head of the animal was then fixed in a mouse holder with a bite bar and two ear inserts (RAPID Biomedical GmbH, Rimpar, Germany). The body temperature was kept at 37.0 ± 0.5 °C by tubing with circulating warm water controlled with a rectal probe. Spontaneous breathing was maintained at 90 ± 20 rpm by adjusting the isoflurane concentration. The respiration rate and body temperature were monitored by a small animal monitor (SA Instruments Inc., Stony Brook, NY, USA).

Cerebral cortex water content

Cortical water content was measured in both WT and *gclm*-KO, at the different time points of interest: P20 (WT: 6, KO: 7); P40 (WT: 6, KO: 5), and P90 (WT: 11, KO: 3) (Fig. S2). A strong age effect was observed (two-way ANOVA, $P < 0.0001$) with a significant decrease between P20 and P40 for both genotypes ($P < 0.0001$), and between P40 and P90 for WT ($P < 0.01$). *Gclm*-KO displayed reduced water content at P40 ($P < 0.05$) in comparison to age-matched WT.

Fig. S1. Example ^{1}H MR spectra (A) and voxel positions for ^{1}H and ^{31}P MRS (B) in a P40 mouse. ^{1}H spectra from a mouse brain depicting creatine + phosphocreatine (Cr + PCr), glucose (Glc), lactate (Lac), alanine (Ala), N-acetylaspartate (NAA), N-acetylaspartylglutamate (NAAG), phosphoetanolamine (PE), phosphocholine (PC), glycerophosphocholine (GPC), ascorbate (Asc), glutathione (GSH), inositol (Ins), taurine (Tau), glutamate (Glu), glutamine (Gln), γ-aminobutyrate (GABA), aspartate (Asp), glycine (Gly) and serine (Ser).

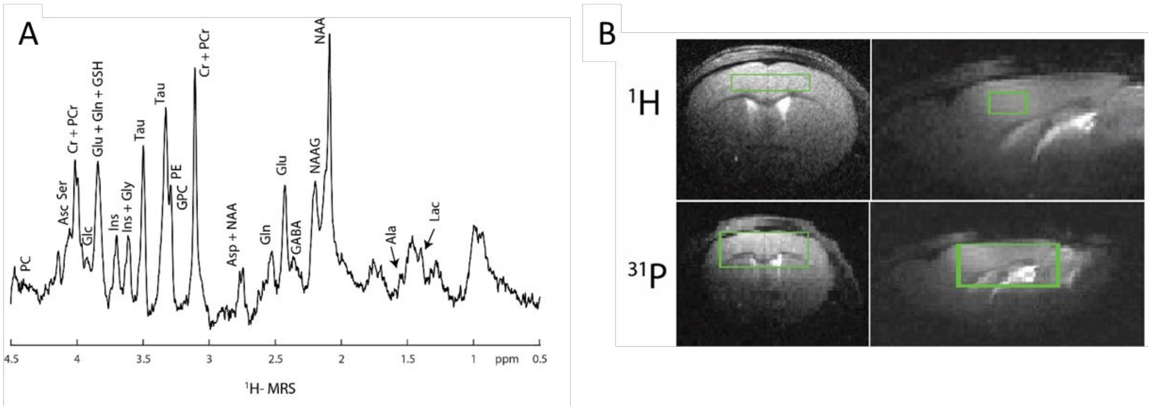

Fig. S2. Age dependent cortex water content (%) in WT and *gclm*-KO mice.

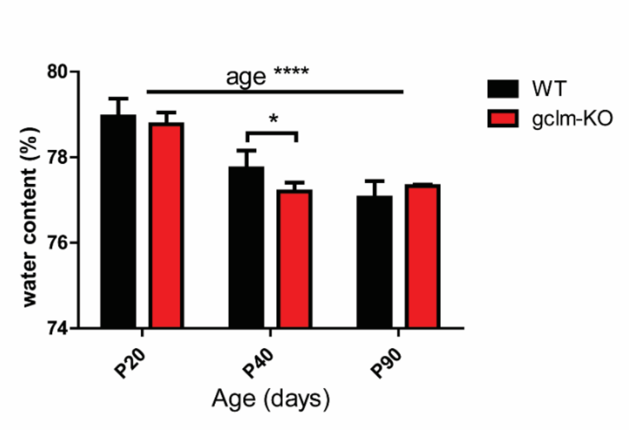

Fig. S3. Concentrations of PME and PDE in *gclm*-KO and WT mouse brain during brain development.

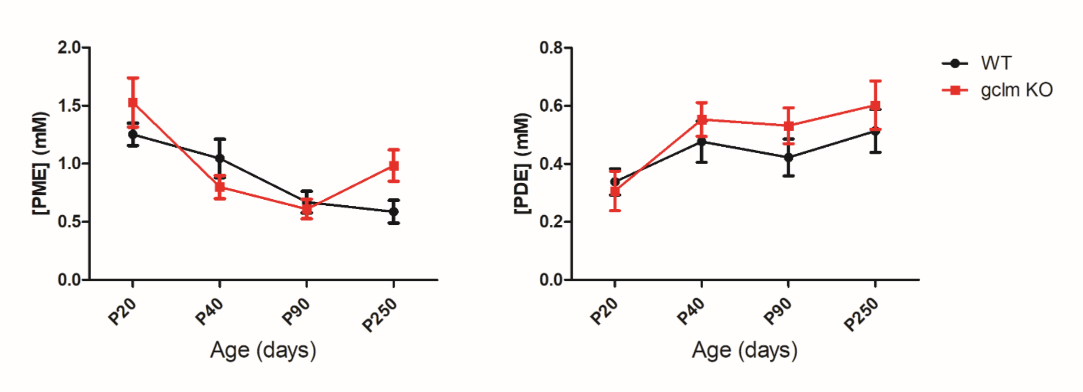

Fig. S4. Concentrations of GSH in *gclm*-KO and WT mouse brain during brain development. Two-way ANOVA (age and genotype) analysis showed significantly lower GSH in *gclm*-KO mice relative to that of WT mice (**** P < 0.0001).

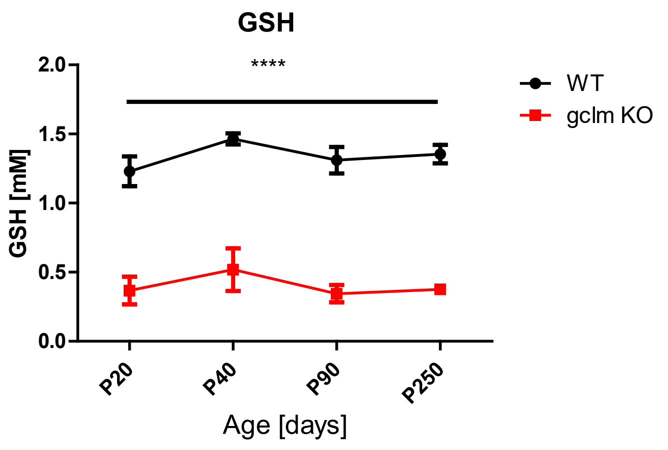

Table 1S. The number of animals for reported metabolites (CRLB < 30%) in each genotype and age group.

	P20		P40		P90		P250	
	WT	*Gclm*-KO	WT	*Gclm*-KO	WT	*Gclm*-KO	WT	*Gclm*-KO
	N	*N*	*N*	*N*	*N*	*N*	*N*	*N*
**Cr**	11	7	8	8	8	8	4	4
**PCr**	11	8	8	8	8	8	4	4
**GABA**	10	8	8	8	7	8	5	4
**Gln**	11	8	8	8	8	8	5	4
**Glu**	11	8	9	8	8	8	5	4
**GSH**	11	6	9	8	8	7	5	3
**Ins**	11	7	8	8	8	8	5	4
**NAA**	11	8	9	8	8	8	5	4
**NAAG**	11	8	8	8	7	8	4	4
**Tau**	11	8	8	8	8	8	5	4
**GPC+PCho**	11	6	9	8	8	8	5	4
**Cr+PCr**	11	8	9	8	8	8	5	4
**Glu+Gln**	11	8	9	8	8	8	5	4
**NAA+NAAG**	11	8	9	8	8	8	5	4
**Pi (int)**	10	10	9	8	7	9	5	4
**α-ATP**	10	10	9	8	8	9	5	4
**β-ATP**	10	10	9	8	8	9	5	4
**γ-ATP**	10	10	9	8	8	9	5	4
**NADH**	10	10	9	8	8	7	5	4
**NAD+**	10	10	9	8	8	9	5	4
**total NAD**	10	10	9	8	8	9	5	4
**PME(PC+PE)**	10	10	9	8	8	8	5	4
**PDE(GPC+GPE)**	10	10	9	8	7	9	5	4
**pH (int)**	10	10	9	8	7	9	5	4
**[Mg^{2+}]**	10	10	9	8	8	9	5	4

Table 2S. Summary of two-way ANOVA (age and genotype) results for all variables. (*) P < 0.05, (**) P < 0.01, (***) P < 0.001, and (****) P < 0.0001.

	Interaction	Genotype	Age
NAD^+	*	**	
NADH			***
NAD^+/NADH	*	*	**
Total NAD			*
GABA			
Gln	*		
Glu	*		*
Gln + Glu	**		
PME/PDE			****
ATP	**	*	****
PCr			****
Pi int			*
pH			****
Mg^{2+}			*

Table 3S. Levels of UDPG, NAD^+, NADH, RR and total NAD obtained with and without inclusion of UDPG in the quantification from summed spectra at P20, P40, P90 and P250.

		with UDPG					without UDPG			
AGE	GENOTYPE	NAD^+	NADH	UDPG	RR	TOTAL NAD	NAD^+	NADH	RR	TOTAL NAD
[DAYS]		[mM]	[mM]	[mM]	[-]	[mM]	[mM]	[mM]	[-]	[mM]
**P20**	WT	0.352	0.194	0.147	2.03	0.545	0.340	0.300	1.13	0.64
	Gclm-KO	0.469	0.103	0.199	5.65	0.572	0.481	0.209	2.30	0.69
**P40**	WT	0.368	0.155	0.185	2.42	0.523	0.354	0.273	1.30	0.627
	Gclm-KO	0.438	0.114	0.168	3.99	0.552	0.447	0.197	2.27	0.644
**P90**	WT	0.36	0.108	0.125	3.19	0.468	0.346	0.203	1.70	0.549
	Gclm-KO	0.622	0.021	0.167	32.75	0.644	0.608	0.122	4.98	0.73
**P250**	WT	0.408	0.043	0.206	8.88	0.451	0.396	0.167	2.37	0.563
	Gclm-KO	0.394	0.144	0.063	3.06	0.538	0.397	0.185	2.15	0.582

Table 4S. Summary of two-way ANOVA (age and genotype) results for UDPG, NAD^+, NADH, and NAD^+/NADH values obtained with inclusion of UDPG in the quantification from individual animal spectra. (*) $P < 0.05$. Significant interaction between age and genotype ($P = 0.038$) was found for UDPG. The *post hoc* analysis using Bonferroni correction for the 4 comparisons between genotypes provided a significant reduction of UDPG levels in *gclm* KO mice at P250 (corrected $p<0.05$), which is consistent as the result of summed spectra (Fig. 2).

	**Interaction**	**Genotype**	**Age**
**NAD^+**		*	
**NADH**			*
**NAD^+/NADH**			*
**UDPG**	*		
